# Expectations in the treatment of delusional disorder: A case report

**DOI:** 10.1192/j.eurpsy.2023.2051

**Published:** 2023-07-19

**Authors:** A. Fuentes Merlos, C. Arroyo del Val, E. X. González Vivero, L. Núñez Cantos, A. Francos Ajona

**Affiliations:** Hospital Clínico San Carlos, Madrid, Spain

## Abstract

**Introduction:**

We present the case of a 43 year-old woman that, following the sudden death of his brother in 2015, begins with symptoms of anxiety, irritability and emotional instability, with a tendency to social isolation. Thereafter, she starts the development of persecutory delusions focused on the work and family environment that evolve into inappropriate behaviors to the point of abandoning her professional life.

**Objectives:**

(1) We will carry out a complete review about persecutory delusions and its relationship with affective disorders, based on the severity of our patient’s case.

(2) We will study the different lines of treatment in delusional disorder (DD) and its course and prognosis in relation to the intervention performed.

**Methods:**

A review of the patient’s history will be conducted, taking into account her biography, clinical evolution and response to the treatments received.

Likewise, a bibliographic review of the available scientific literature in relation to DD treatment strategies will be carried out.

**Results:**

(1) DD is more common in middle-aged women. People who tend to be socially isolated are more likely to develop DD.

(2) Acute onset, in young women with identifiable precipitating factors, suggests a better prognosis.

(3) In the long term, 50% of patients recover and a further 20% experience some improvement.

(4) The combination of antipsychotic medications and psychological therapies such as cognitive behavioral therapy (CBT) is fundamental in the management of DD patients.

**Conclusions:**

The prognosis for patients with DD varies depending on various factors, including the type and severity of the delusional ideas, and their own life circumstances. It is often possible to eliminate the behavioral alterations derived from the DD, allowing the patient to function normally. However, delusions often persist and become encapsulated.

**Disclosure of Interest:**

None Declared

